# 9-[(*E*)-2-(4,4,5,5-Tetra­methyl-1,3,2-dioxa­borolan-2-yl)ethen­yl]-9*H*-carbazole

**DOI:** 10.1107/S1600536811052470

**Published:** 2011-12-10

**Authors:** Yuki Hatayama, Tsunehisa Okuno

**Affiliations:** a Department of Material Science and Chemistry, Wakayama University, Sakaedani, Wakayama 640-8510, Japan

## Abstract

The title compound, C_20_H_22_BNO_2_, is a simple olefinic compound which carries both B and N atoms that are *trans* to one another. The π-conjugated system of the compound is considered to be isoelectronic with 1,3-butadiene. There are two independent mol­ecules in the asymmetric unit in which the environments around the boron atoms are essentially planar (r.m.s. deviations of 0.0032 and 0.0021 Å for the BO_2_C planes). The dihedral angles of the olefinic planes with the boron planes are 5.70 (11) and 9.74 (9)°, respectively, while the dihedral angles of the olefinic planes with the carbazole planes are 19.37 (3) and 10.74 (6)°. These dihedral angles are consistent with those in 9-ethenylcarbazole and an ethenylboronic ester derivative. The N—C*sp* ^2^, B—C*sp* ^2^ and C=C bond lengths suggest that the contribution of the canonical structure can be described as N^+^=C—C=B^−^.

## Related literature

For the related structure of 9-ethenylcarbazole, see: Tsutsui *et al.* (1976 ▶); Tian *et al.* (2006 ▶). For the related structure of a ethenyl-boronic ester derivative, see: Clark *et al.* (2004 ▶). For the preparation of the title compound, see: Geier *et al.* (2009 ▶).
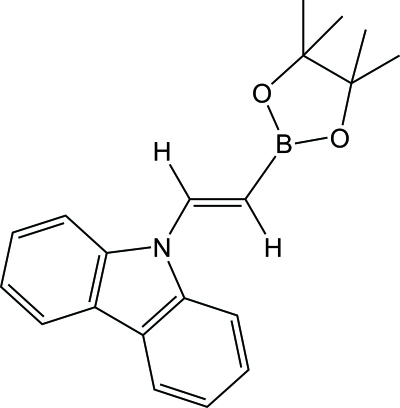


## Experimental

### 

#### Crystal data

C_20_H_22_BNO_2_
*M* *_r_* = 319.21
Triclinic, 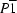

*a* = 11.616 (3) Å
*b* = 12.962 (3) Å
*c* = 12.990 (3) Å
α = 83.620 (11)°
β = 85.632 (11)°
γ = 64.647 (6)°
*V* = 1755.6 (8) Å^3^
*Z* = 4
Mo *K*α radiation
μ = 0.08 mm^−1^
*T* = 93 K
0.19 × 0.07 × 0.03 mm

#### Data collection

Rigaku Saturn724+ diffractometer
Absorption correction: numerical (*NUMABS*; Rigaku, 1999 ▶) *T*_min_ = 0.990, *T*_max_ = 0.998
12732 measured reflections
6408 independent reflections
4117 reflections with *F* ^2^ > 2σ(*F* ^2^)
*R*_int_ = 0.035

#### Refinement

*R*[*F* ^2^ > 2σ(*F* ^2^)] = 0.053
*wR*(*F* ^2^) = 0.137
*S* = 1.01
6406 reflections
433 parameters
H-atom parameters constrained
Δρ_max_ = 0.23 e Å^−3^
Δρ_min_ = −0.22 e Å^−3^

### 

Data collection: *CrystalClear* (Rigaku, 2008 ▶); cell refinement: *CrystalClear*; data reduction: *CrystalClear*; program(s) used to solve structure: *SIR92* (Altomare *et al.*, 1994 ▶); program(s) used to refine structure: *SHELXL97* (Sheldrick, 2008 ▶); molecular graphics: *ORTEP-3* (Farrugia, 1997 ▶); software used to prepare material for publication: *CrystalStructure* (Rigaku, 2010 ▶).

## Supplementary Material

Crystal structure: contains datablock(s) global, I. DOI: 10.1107/S1600536811052470/ff2046sup1.cif

Structure factors: contains datablock(s) I. DOI: 10.1107/S1600536811052470/ff2046Isup2.hkl

Supplementary material file. DOI: 10.1107/S1600536811052470/ff2046Isup3.cml

Additional supplementary materials:  crystallographic information; 3D view; checkCIF report

## Figures and Tables

**Table 1:** Selected bond lengths (Å)

B1—C14	1.537 (3)
B2—C34	1.537 (4)
N1—C13	1.396 (3)
N2—C33	1.392 (3)
C13—C14	1.336 (4)
C33—C34	1.334 (4)

**Fig. 1.**
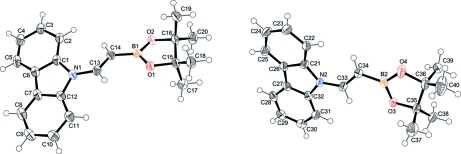
The asymmetric unit of the title compound with atom-numbering scheme. Displacement ellipsoids are drawn at the 50% probability level and H atoms are shown as small spheres.

## supplementary crystallographic information

### Comment

Boron and nitrogen atoms are known to be isoelectronic with carbocation and carbanion, respectively. Several isoelectronic structures have been prepared by replacing C=C bond of aromatic systems with B—N bond. In order to expand the varieties of isoelectronic structures by utilizing combination of boron and nitrogen atoms, an insertion of C=C bond into B—N bond is quite important. The title compound, C_20_H_22_BNO_2_, is a simple olefinic compound which carries both boron and nitrogen atoms in a *trans* position. The π-conjugated system is considered to be isoelectronic with 1,3-butadiene.

There are two independent molecules in the asymmetric unit and their structures are similar (Fig.1). The environments around the boron atoms, the B1/O1/O2/C14 plane (r.m.s. deviation 0.0032 Å and the B2/O3/O4/C34 plane (r.m.s. deviation 0.0021 Å), are planar. The dihedral angles of the C13/H13/C14/H14 plane (r.m.s. deviation 0.0095 Å with the N1/C1—C12 plane (r.m.s. deviation 0.0191 Å and the B1/O1/O2/C14 plane are 19.37 (3)° and 5.70 (11)°, respectively. The dihedral angles of the C33/H33/C34/H34 plane (r.m.s. deviation 0.0111 Å with the N2/C21—C32 plane (r.m.s. deviation 0.0376 Å and the B2/O3/O4/C34 plane are 10.74 (6)° and 9.74 (9)°, respectively. These dihedral angles are consistent with those in 9-ethenylcarbazole (Tsutsui *et al.*, 1976; Tian *et al.*, 2006) and a ethenylboronic ester derivative (Clark *et al.*, 2004). The bond distances of N1—C13 and N2—C33 show a tendency to be shorten compared with N-C*sp*^2^ distance of 9-ethenylcarbazole (1.398 (8)–1.425 (7) Å). The bond distances of B1—C14 and B2—C34 are also tend to be short compared with B-C*sp*^2^ distance of the reported ethenylboronic ester derivative (1.544 (5) Å). The bond distances of C13—C14 and C33—C34 display a tendency to be elongated compared with C=C bond distances of 9-ethenylcarbazole (1.231 (8)–1.2861 (3) Å or a ethenylboronic ester derivative (1.325 (5) Å). These tendencies were well explained by considering the canonical structure of the π-conjugated system such as N^+^=C—C=B^-^.

Although a boron atom without steric protection shows intermolecular contact with a lone pair of another atom in many cases, the spatial contact between the compounds could not be detected. This is presumably because the vacant *p*-orbital of boron atom is satisfied with the lone pairs of oxygen atoms and/or π electrons of the C=C bond.

### Experimental

The title compound was prepared according to a published procedure (Geier *et al.*, 2009). The purification of the compound was performed by gel permeation chromatography (GPC). The single crystals with sufficient quality for X-ray analysis were obtained by concentration of a hexane solution in a refrigerator.

### Refinement

The C-bound H atoms were placed at ideal positions and were refined as riding on their parent C atoms. *U*_iso_(H) values of the H atoms were set at 1.2*U*_eq_(parent atom).

### Crystal data

**Table:**

C_20_H_22_BNO_2_	*Z* = 4
*M**_r_* = 319.21	*F*(000) = 680.00
Triclinic, *P*1	*D*_x_ = 1.208 Mg m^−^^3^
Hall symbol: -P 1	Mo *K*α radiation, λ = 0.71075 Å
*a* = 11.616 (3) Å	Cell parameters from 4367 reflections
*b* = 12.962 (3) Å	θ = 1.7–30.9°
*c* = 12.990 (3) Å	µ = 0.08 mm^−^^1^
α = 83.620 (11)°	*T* = 93 K
β = 85.632 (11)°	Prism, colourless
γ = 64.647 (6)°	0.19 × 0.07 × 0.03 mm
*V* = 1755.6 (8) Å^3^	

### Data collection

**Table:**

Rigaku Saturn724+ diffractometer	4117 reflections with *F*^2^ > 2σ(*F*^2^)
Detector resolution: 29.257 pixels mm^-1^	*R*_int_ = 0.035
ω scans	θ_max_ = 25.5°
Absorption correction: numerical (*NUMABS*; Rigaku, 1999)	*h* = −14→9
*T*_min_ = 0.990, *T*_max_ = 0.998	*k* = −15→15
12732 measured reflections	*l* = −14→15
6408 independent reflections	

### Refinement

**Table:**

Refinement on *F*^2^	Secondary atom site location: difference Fourier map
*R*[*F*^2^ > 2σ(*F*^2^)] = 0.053	Hydrogen site location: inferred from neighbouring sites
*wR*(*F*^2^) = 0.137	H-atom parameters constrained
*S* = 1.01	*w* = 1/[σ^2^(*F*_o_^2^) + (0.068*P*)^2^] where *P* = (*F*_o_^2^ + 2*F*_c_^2^)/3
6406 reflections	(Δ/σ)_max_ < 0.001
433 parameters	Δρ_max_ = 0.23 e Å^−^^3^
0 restraints	Δρ_min_ = −0.22 e Å^−^^3^
Primary atom site location: structure-invariant direct methods	

### Special details

**Table:**

Refinement. Refinement was performed using all reflections except for 2 with very negative *F*^2^. The weighted *R*-factor (*wR*) and goodness of fit (*S*) are based on *F*^2^. *R*-factor (gt) are based on *F*. The threshold expression of *F*^2^ > 2.0 σ(*F*^2^) is used only for calculating *R*-factor (gt).

### Fractional atomic coordinates and isotropic or equivalent isotropic displacement parameters (Å^2^)

**Table:**

	*x*	*y*	*z*	*U*_iso_*/*U*_eq_	
O1	1.19650 (14)	0.80244 (12)	0.27030 (11)	0.0288 (4)	
O2	1.05702 (13)	0.72769 (12)	0.23902 (11)	0.0276 (4)	
O3	0.73428 (13)	0.89367 (12)	0.10030 (10)	0.0267 (4)	
O4	0.65878 (15)	0.75895 (15)	0.08875 (13)	0.0446 (5)	
N1	0.89819 (16)	1.15426 (15)	0.21620 (13)	0.0266 (5)	
N2	0.45751 (16)	1.05543 (15)	0.34418 (12)	0.0251 (4)	
C1	0.7867 (2)	1.20195 (18)	0.15851 (16)	0.0266 (5)	
C2	0.7364 (2)	1.15124 (19)	0.09656 (16)	0.0296 (6)	
C3	0.6224 (2)	1.22080 (19)	0.04871 (17)	0.0336 (6)	
C4	0.5611 (3)	1.3375 (2)	0.06069 (18)	0.0381 (6)	
C5	0.6129 (3)	1.3884 (2)	0.11964 (18)	0.0359 (6)	
C6	0.7264 (2)	1.32114 (18)	0.16925 (16)	0.0296 (6)	
C7	0.8042 (2)	1.34695 (18)	0.23469 (17)	0.0300 (6)	
C8	0.7935 (3)	1.4481 (2)	0.27136 (18)	0.0380 (6)	
C9	0.8881 (3)	1.4439 (2)	0.33207 (19)	0.0407 (6)	
C10	0.9931 (3)	1.3404 (2)	0.35646 (18)	0.0373 (6)	
C11	1.0044 (3)	1.2395 (2)	0.32233 (17)	0.0325 (6)	
C12	0.9099 (2)	1.24341 (18)	0.26120 (16)	0.0274 (5)	
C13	0.9859 (2)	1.03972 (18)	0.23079 (15)	0.0263 (5)	
C14	0.9701 (2)	0.94585 (18)	0.21762 (15)	0.0260 (5)	
C15	1.2589 (2)	0.68006 (18)	0.30464 (17)	0.0293 (5)	
C16	1.1828 (2)	0.63081 (18)	0.24721 (17)	0.0301 (6)	
C17	1.2405 (3)	0.6684 (2)	0.42173 (17)	0.0405 (7)	
C18	1.3987 (2)	0.6370 (2)	0.27360 (19)	0.0364 (6)	
C19	1.2325 (3)	0.6055 (2)	0.13647 (18)	0.0359 (6)	
C20	1.1668 (3)	0.52875 (19)	0.30432 (19)	0.0400 (6)	
C21	0.35972 (19)	1.03528 (18)	0.40093 (15)	0.0259 (5)	
C22	0.3077 (2)	0.95879 (19)	0.38847 (16)	0.0283 (5)	
C23	0.2077 (2)	0.9603 (2)	0.45537 (17)	0.0333 (6)	
C24	0.1605 (3)	1.0353 (2)	0.53272 (17)	0.0367 (6)	
C25	0.2107 (2)	1.1126 (2)	0.54471 (17)	0.0333 (6)	
C26	0.3101 (2)	1.11383 (18)	0.47789 (15)	0.0263 (5)	
C27	0.3769 (2)	1.18617 (18)	0.46632 (15)	0.0256 (5)	
C28	0.3674 (2)	1.27887 (19)	0.51777 (17)	0.0318 (6)	
C29	0.4455 (3)	1.33272 (19)	0.48543 (17)	0.0338 (6)	
C30	0.5321 (3)	1.2958 (2)	0.40222 (17)	0.0332 (6)	
C31	0.5438 (2)	1.20373 (19)	0.35044 (17)	0.0305 (6)	
C32	0.4653 (2)	1.14974 (18)	0.38344 (15)	0.0253 (5)	
C33	0.54010 (19)	0.99494 (19)	0.26653 (15)	0.0264 (5)	
C34	0.5570 (2)	0.89644 (18)	0.23134 (16)	0.0292 (5)	
C35	0.7890 (2)	0.84178 (19)	0.00247 (15)	0.0292 (5)	
C36	0.7708 (3)	0.7292 (3)	0.01803 (19)	0.0399 (6)	
C37	0.7120 (3)	0.9265 (3)	−0.08385 (18)	0.0499 (8)	
C38	0.9265 (2)	0.8232 (2)	−0.00862 (17)	0.0326 (6)	
C39	0.8800 (3)	0.6309 (2)	0.0738 (3)	0.0529 (8)	
C40	0.7429 (3)	0.6927 (3)	−0.0810 (3)	0.0710 (10)	
B1	1.0760 (3)	0.8250 (2)	0.24176 (18)	0.0263 (6)	
B2	0.6507 (3)	0.8488 (3)	0.1401 (2)	0.0285 (6)	
H2	0.7785	1.0717	0.0873	0.0355*	
H3	0.5856	1.1879	0.0069	0.0403*	
H4	0.4826	1.3828	0.0279	0.0457*	
H5	0.5715	1.4686	0.1262	0.0430*	
H8	0.7222	1.5186	0.2548	0.0456*	
H9	0.8817	1.5122	0.3576	0.0488*	
H10	1.0579	1.3397	0.3973	0.0448*	
H11	1.0752	1.1691	0.3403	0.0390*	
H13	1.0674	1.0269	0.2529	0.0316*	
H14	0.8916	0.9539	0.1928	0.0312*	
H17A	1.1492	0.6998	0.4401	0.0486*	
H17B	1.2795	0.7104	0.4533	0.0486*	
H17C	1.2810	0.5872	0.4473	0.0486*	
H18A	1.4363	0.6791	0.3070	0.0437*	
H18B	1.4079	0.6491	0.1982	0.0437*	
H18C	1.4426	0.5551	0.2955	0.0437*	
H19A	1.2387	0.6733	0.0996	0.0430*	
H19B	1.1738	0.5863	0.1009	0.0430*	
H19C	1.3170	0.5409	0.1378	0.0430*	
H20A	1.1106	0.5093	0.2657	0.0480*	
H20B	1.1292	0.5481	0.3736	0.0480*	
H20C	1.2502	0.4631	0.3105	0.0480*	
H22	0.3395	0.9072	0.3359	0.0339*	
H23	0.1709	0.9088	0.4480	0.0399*	
H24	0.0930	1.0335	0.5779	0.0441*	
H25	0.1780	1.1640	0.5975	0.0399*	
H28	0.3081	1.3044	0.5742	0.0382*	
H29	0.4402	1.3955	0.5201	0.0405*	
H30	0.5841	1.3346	0.3806	0.0399*	
H31	0.6036	1.1784	0.2943	0.0366*	
H33	0.5926	1.0291	0.2334	0.0317*	
H34	0.5108	0.8554	0.2630	0.0351*	
H37A	0.7186	0.9992	−0.0823	0.0598*	
H37B	0.6225	0.9395	−0.0742	0.0598*	
H37C	0.7452	0.8955	−0.1508	0.0598*	
H38A	0.9737	0.7721	0.0500	0.0391*	
H38B	0.9314	0.8970	−0.0097	0.0391*	
H38C	0.9638	0.7885	−0.0734	0.0391*	
H39A	0.8581	0.5657	0.0900	0.0635*	
H39B	0.8951	0.6555	0.1381	0.0635*	
H39C	0.9572	0.6081	0.0291	0.0635*	
H40A	0.6693	0.7558	−0.1138	0.0852*	
H40B	0.7239	0.6258	−0.0643	0.0852*	
H40C	0.8173	0.6728	−0.1286	0.0852*	

### Atomic displacement parameters (Å^2^)

**Table:**

	*U*^11^	*U*^22^	*U*^33^	*U*^12^	*U*^13^	*U*^23^
O1	0.0296 (9)	0.0213 (8)	0.0343 (9)	−0.0095 (7)	−0.0038 (7)	−0.0006 (7)
O2	0.0256 (8)	0.0199 (8)	0.0345 (9)	−0.0073 (7)	0.0004 (7)	−0.0021 (7)
O3	0.0296 (9)	0.0262 (8)	0.0223 (8)	−0.0095 (7)	0.0040 (6)	−0.0065 (6)
O4	0.0408 (10)	0.0484 (11)	0.0571 (11)	−0.0285 (9)	0.0249 (8)	−0.0320 (9)
N1	0.0274 (11)	0.0209 (10)	0.0293 (10)	−0.0089 (8)	0.0045 (8)	−0.0031 (8)
N2	0.0243 (10)	0.0263 (10)	0.0227 (10)	−0.0088 (8)	0.0027 (8)	−0.0052 (8)
C1	0.0229 (12)	0.0233 (12)	0.0290 (12)	−0.0070 (10)	0.0068 (10)	0.0000 (10)
C2	0.0292 (13)	0.0242 (12)	0.0307 (12)	−0.0081 (10)	0.0011 (10)	0.0015 (10)
C3	0.0308 (14)	0.0309 (13)	0.0365 (13)	−0.0120 (11)	0.0013 (11)	0.0018 (11)
C4	0.0288 (13)	0.0353 (14)	0.0404 (14)	−0.0071 (12)	0.0030 (11)	0.0053 (12)
C5	0.0311 (14)	0.0244 (13)	0.0412 (14)	−0.0039 (11)	0.0072 (11)	0.0023 (11)
C6	0.0278 (13)	0.0235 (12)	0.0332 (13)	−0.0085 (10)	0.0098 (10)	−0.0021 (10)
C7	0.0294 (13)	0.0230 (12)	0.0355 (13)	−0.0107 (10)	0.0111 (10)	−0.0038 (10)
C8	0.0392 (15)	0.0267 (13)	0.0451 (15)	−0.0128 (12)	0.0122 (12)	−0.0057 (11)
C9	0.0479 (16)	0.0291 (14)	0.0484 (15)	−0.0198 (13)	0.0125 (13)	−0.0115 (12)
C10	0.0398 (15)	0.0365 (15)	0.0400 (14)	−0.0201 (12)	0.0047 (11)	−0.0081 (11)
C11	0.0356 (14)	0.0287 (13)	0.0335 (13)	−0.0151 (11)	0.0059 (11)	−0.0032 (10)
C12	0.0319 (13)	0.0245 (12)	0.0274 (12)	−0.0144 (11)	0.0082 (10)	−0.0044 (10)
C13	0.0243 (12)	0.0249 (12)	0.0257 (12)	−0.0074 (10)	0.0011 (9)	−0.0004 (9)
C14	0.0265 (12)	0.0236 (12)	0.0260 (12)	−0.0088 (10)	0.0010 (9)	−0.0032 (9)
C15	0.0290 (13)	0.0192 (11)	0.0371 (13)	−0.0075 (10)	−0.0036 (10)	−0.0016 (10)
C16	0.0247 (12)	0.0188 (11)	0.0426 (14)	−0.0050 (10)	−0.0029 (10)	−0.0028 (10)
C17	0.0459 (16)	0.0348 (14)	0.0376 (14)	−0.0137 (13)	−0.0070 (12)	−0.0006 (11)
C18	0.0304 (13)	0.0275 (13)	0.0482 (15)	−0.0086 (11)	−0.0053 (11)	−0.0036 (11)
C19	0.0299 (13)	0.0299 (13)	0.0462 (14)	−0.0091 (11)	−0.0004 (11)	−0.0124 (11)
C20	0.0401 (15)	0.0267 (13)	0.0546 (16)	−0.0161 (12)	−0.0084 (12)	0.0045 (12)
C21	0.0199 (11)	0.0299 (12)	0.0239 (11)	−0.0075 (10)	0.0004 (9)	−0.0006 (10)
C22	0.0267 (12)	0.0278 (12)	0.0274 (12)	−0.0089 (10)	0.0013 (10)	−0.0032 (10)
C23	0.0319 (13)	0.0362 (14)	0.0316 (13)	−0.0152 (11)	0.0014 (11)	−0.0009 (11)
C24	0.0331 (14)	0.0463 (16)	0.0288 (13)	−0.0170 (12)	0.0098 (10)	−0.0010 (11)
C25	0.0340 (14)	0.0374 (14)	0.0252 (12)	−0.0123 (12)	0.0035 (10)	−0.0049 (10)
C26	0.0262 (12)	0.0264 (12)	0.0211 (11)	−0.0060 (10)	−0.0029 (9)	−0.0009 (9)
C27	0.0250 (12)	0.0254 (12)	0.0216 (11)	−0.0058 (10)	−0.0044 (9)	−0.0010 (9)
C28	0.0317 (13)	0.0283 (13)	0.0287 (12)	−0.0052 (11)	−0.0046 (10)	−0.0044 (10)
C29	0.0383 (14)	0.0272 (13)	0.0335 (13)	−0.0093 (11)	−0.0110 (11)	−0.0052 (10)
C30	0.0366 (14)	0.0323 (14)	0.0337 (13)	−0.0173 (12)	−0.0074 (11)	0.0012 (11)
C31	0.0284 (13)	0.0327 (13)	0.0287 (12)	−0.0115 (11)	0.0021 (10)	−0.0036 (10)
C32	0.0255 (12)	0.0250 (12)	0.0237 (11)	−0.0081 (10)	−0.0034 (9)	−0.0036 (9)
C33	0.0218 (12)	0.0315 (13)	0.0229 (11)	−0.0089 (10)	0.0008 (9)	−0.0020 (10)
C34	0.0282 (13)	0.0270 (13)	0.0310 (12)	−0.0110 (10)	0.0047 (10)	−0.0037 (10)
C35	0.0307 (13)	0.0331 (13)	0.0222 (11)	−0.0114 (11)	0.0043 (10)	−0.0082 (10)
C36	0.0369 (14)	0.0416 (15)	0.0479 (15)	−0.0214 (12)	0.0202 (12)	−0.0250 (12)
C37	0.0461 (17)	0.0581 (18)	0.0305 (14)	−0.0076 (14)	−0.0037 (12)	−0.0037 (13)
C38	0.0333 (13)	0.0388 (14)	0.0266 (12)	−0.0167 (11)	0.0081 (10)	−0.0069 (10)
C39	0.0555 (18)	0.0281 (14)	0.0670 (19)	−0.0143 (13)	0.0294 (14)	−0.0077 (13)
C40	0.065 (2)	0.096 (3)	0.080 (3)	−0.054 (2)	0.0319 (17)	−0.063 (2)
B1	0.0312 (15)	0.0260 (14)	0.0221 (13)	−0.0129 (12)	0.0029 (11)	−0.0027 (11)
B2	0.0243 (14)	0.0281 (14)	0.0330 (14)	−0.0108 (12)	−0.0003 (11)	−0.0041 (11)

### Geometric parameters (Å, °)

**Table:**

O1—C15	1.466 (3)	C35—C36	1.551 (5)
O1—B1	1.373 (4)	C35—C37	1.517 (3)
O2—C16	1.465 (3)	C35—C38	1.507 (4)
O2—B1	1.376 (4)	C36—C39	1.519 (4)
O3—C35	1.474 (3)	C36—C40	1.522 (5)
O3—B2	1.374 (4)	C2—H2	0.950
O4—C36	1.467 (3)	C3—H3	0.950
O4—B2	1.371 (4)	C4—H4	0.950
N1—C1	1.407 (3)	C5—H5	0.950
N1—C12	1.412 (4)	C8—H8	0.950
N1—C13	1.396 (3)	C9—H9	0.950
N2—C21	1.409 (3)	C10—H10	0.950
N2—C32	1.415 (4)	C11—H11	0.950
N2—C33	1.392 (3)	C13—H13	0.950
C1—C2	1.393 (4)	C14—H14	0.950
C1—C6	1.415 (3)	C17—H17A	0.980
C2—C3	1.390 (3)	C17—H17B	0.980
C3—C4	1.391 (4)	C17—H17C	0.980
C4—C5	1.381 (5)	C18—H18A	0.980
C5—C6	1.391 (3)	C18—H18B	0.980
C6—C7	1.446 (4)	C18—H18C	0.980
C7—C8	1.397 (4)	C19—H19A	0.980
C7—C12	1.406 (3)	C19—H19B	0.980
C8—C9	1.380 (5)	C19—H19C	0.980
C9—C10	1.397 (3)	C20—H20A	0.980
C10—C11	1.378 (4)	C20—H20B	0.980
C11—C12	1.383 (4)	C20—H20C	0.980
C13—C14	1.336 (4)	C22—H22	0.950
C14—B1	1.537 (3)	C23—H23	0.950
C15—C16	1.560 (4)	C24—H24	0.950
C15—C17	1.520 (3)	C25—H25	0.950
C15—C18	1.512 (4)	C28—H28	0.950
C16—C19	1.525 (4)	C29—H29	0.950
C16—C20	1.519 (4)	C30—H30	0.950
C21—C22	1.392 (4)	C31—H31	0.950
C21—C26	1.416 (3)	C33—H33	0.950
C22—C23	1.391 (4)	C34—H34	0.950
C23—C24	1.390 (4)	C37—H37A	0.980
C24—C25	1.385 (5)	C37—H37B	0.980
C25—C26	1.395 (4)	C37—H37C	0.980
C26—C27	1.442 (4)	C38—H38A	0.980
C27—C28	1.399 (4)	C38—H38B	0.980
C27—C32	1.402 (3)	C38—H38C	0.980
C28—C29	1.380 (4)	C39—H39A	0.980
C29—C30	1.395 (3)	C39—H39B	0.980
C30—C31	1.385 (4)	C39—H39C	0.980
C31—C32	1.388 (4)	C40—H40A	0.980
C33—C34	1.334 (4)	C40—H40B	0.980
C34—B2	1.537 (4)	C40—H40C	0.980
			
O1···C13	3.017 (3)	C26···H10^iv^	3.2689
O1···C19	3.110 (4)	C26···H11^iv^	3.1521
O2···C17	3.119 (3)	C27···H10^iv^	3.5052
O3···C33	2.971 (3)	C28···H9^xi^	3.4471
O3···C39	3.134 (3)	C28···H18A^x^	3.5790
O4···C37	3.159 (4)	C28···H20C^viii^	3.3390
N1···C5	3.598 (3)	C28···H34^iii^	3.2475
C1···C4	2.764 (3)	C29···H17B^x^	3.1483
C1···C14	3.126 (3)	C29···H17C^viii^	3.0266
C2···C5	2.822 (4)	C29···H18A^x^	3.0783
C2···C13	3.187 (3)	C29···H18C^viii^	3.5697
C2···C14	3.235 (3)	C29···H20C^viii^	3.1325
C3···C6	2.773 (4)	C30···H17B^x^	2.9601
C5···C8	3.353 (5)	C30···H18C^viii^	3.2391
C7···C10	2.768 (4)	C30···H20C^viii^	3.2835
C8···C11	2.816 (3)	C33···H25^iii^	3.5184
C9···C12	2.756 (4)	C33···H37B^v^	3.0667
C11···C13	3.060 (4)	C33···H37C^v^	3.3930
C17···C20	2.911 (5)	C33···H40A^v^	3.5877
C17···B1	3.089 (4)	C34···H28^iii^	3.3559
C18···C19	2.903 (4)	C34···H37A^v^	3.5331
C18···C20	3.513 (5)	C34···H37B^v^	2.9875
C18···B1	3.514 (4)	C34···H37C^v^	3.5375
C19···B1	3.054 (4)	C35···H2	3.2424
C20···B1	3.534 (4)	C35···H14	3.5176
C21···C24	2.772 (4)	C37···H2	3.3948
C21···C34	3.150 (3)	C37···H3	3.3753
C22···C25	2.827 (4)	C37···H13^ii^	3.4537
C22···C33	3.207 (4)	C38···H2	3.2725
C22···C34	3.266 (3)	C38···H13^ii^	3.5449
C23···C26	2.769 (5)	C38···H14	3.1802
C25···C28	3.343 (5)	C38···H19B	3.4358
C27···C30	2.768 (4)	C39···H8^vi^	3.4457
C28···C31	2.820 (3)	C39···H19B	3.2488
C29···C32	2.760 (4)	C39···H20A	3.5219
C31···C33	3.046 (4)	C39···H39C^xiii^	3.2368
C37···C40	2.892 (6)	C40···H3^v^	3.5481
C37···B2	3.103 (4)	C40···H4^v^	3.1717
C38···C39	2.850 (5)	C40···H19B^xiii^	3.3541
C38···C40	3.471 (6)	C40···H19C^xiii^	3.5448
C38···B2	3.521 (4)	C40···H20A^xiii^	3.5302
C39···B2	3.093 (4)	B1···H23^i^	3.4196
C40···B2	3.529 (5)	B1···H24^iii^	3.1429
O1···C22^i^	3.372 (4)	B1···H25^iii^	3.4434
O1···C23^i^	3.370 (4)	B1···H38A	3.1009
O2···C38	3.526 (3)	B2···H37B^v^	3.2813
O3···C2	3.344 (4)	H2···O3	2.5567
O3···C14	3.569 (4)	H2···C35	3.2424
N1···C38^ii^	3.317 (3)	H2···C37	3.3948
N2···C26^iii^	3.492 (3)	H2···C38	3.2725
C2···O3	3.344 (4)	H2···H31	3.2950
C11···C24^i^	3.578 (3)	H2···H33	2.9556
C13···C38^ii^	3.533 (4)	H2···H37A	2.7348
C14···O3	3.569 (4)	H2···H38B	2.5925
C14···C24^iii^	3.462 (3)	H3···O3	3.5567
C21···C27^iii^	3.572 (3)	H3···O4^v^	2.9576
C21···C32^iii^	3.596 (3)	H3···C37	3.3753
C22···O1^iv^	3.372 (4)	H3···C40^v^	3.5481
C23···O1^iv^	3.370 (4)	H3···H18B^ii^	3.2288
C24···C11^iv^	3.578 (3)	H3···H19A^ii^	3.3887
C24···C14^iii^	3.462 (3)	H3···H33	3.3831
C26···N2^iii^	3.492 (3)	H3···H37A	2.6147
C27···C21^iii^	3.572 (3)	H3···H37B	3.3418
C32···C21^iii^	3.596 (3)	H3···H37B^v^	3.4791
C34···C37^v^	3.535 (4)	H3···H40A^v^	2.9959
C37···C34^v^	3.535 (4)	H3···H40B^v^	3.4447
C38···O2	3.526 (3)	H4···O4^v^	3.4571
C38···N1^ii^	3.317 (3)	H4···C4^vii^	3.5083
C38···C13^ii^	3.533 (4)	H4···C5^vii^	3.1527
O1···H13	2.6326	H4···C19^viii^	3.4437
O1···H14	3.4004	H4···C40^v^	3.1717
O1···H17A	2.5927	H4···H4^vii^	3.2307
O1···H17B	2.5956	H4···H5^vii^	2.5326
O1···H17C	3.2569	H4···H18B^ii^	3.1088
O1···H18A	2.6000	H4···H19A^ii^	3.3451
O1···H18B	2.5969	H4···H19B^viii^	3.5571
O1···H18C	3.2583	H4···H19C^viii^	2.6059
O1···H19A	2.8139	H4···H19C^ii^	3.4309
O2···H14	2.7591	H4···H40A^v^	3.0916
O2···H17A	2.8254	H4···H40B^v^	2.4566
O2···H19A	2.5804	H5···C4^vii^	3.2378
O2···H19B	2.5790	H5···C18^viii^	3.0086
O2···H19C	3.2483	H5···C19^viii^	3.5579
O2···H20A	2.6133	H5···H4^vii^	2.5326
O2···H20B	2.6130	H5···H18A^viii^	3.5520
O2···H20C	3.2682	H5···H18B^viii^	2.5229
O3···H33	2.5590	H5···H18C^viii^	2.6356
O3···H34	3.3888	H5···H19C^viii^	2.6893
O3···H37A	2.5809	H5···H19C^ii^	3.5690
O3···H37B	2.5816	H5···H30	3.5397
O3···H37C	3.2528	H8···O4^ix^	3.4179
O3···H38A	2.6159	H8···C18^viii^	3.3972
O3···H38B	2.6184	H8···C39^ix^	3.4457
O3···H38C	3.2734	H8···H18A^viii^	3.1419
O3···H39B	2.8506	H8···H18B^viii^	3.4023
O4···H34	2.7989	H8···H18C^viii^	3.0879
O4···H37B	2.8849	H8···H28^xi^	3.2629
O4···H39A	2.5820	H8···H29^xi^	3.3817
O4···H39B	2.5823	H8···H30	3.5972
O4···H39C	3.2502	H8···H39A^ix^	2.7319
O4···H40A	2.6288	H8···H39B^ix^	3.3861
O4···H40B	2.6302	H9···C20^ix^	3.4233
O4···H40C	3.2800	H9···C28^xi^	3.4471
N1···H2	2.8113	H9···H17C^x^	3.5047
N1···H11	2.7883	H9···H20A^ix^	2.8175
N1···H14	2.6814	H9···H20B^ix^	3.1279
N2···H22	2.8125	H9···H20B^x^	3.4972
N2···H31	2.7862	H9···H28^xi^	2.6427
N2···H34	2.7119	H9···H39A^ix^	3.4756
C1···H3	3.2430	H9···H39B^ix^	3.2565
C1···H5	3.2876	H10···C20^ix^	3.2907
C1···H13	3.3075	H10···C25^i^	3.2043
C1···H14	2.9029	H10···C26^i^	3.2689
C2···H4	3.2747	H10···C27^i^	3.5052
C2···H14	2.6526	H10···H17A^x^	3.2488
C3···H5	3.2678	H10···H20A^ix^	2.8790
C4···H2	3.2842	H10···H20B^ix^	3.1254
C5···H3	3.2595	H10···H20C^ix^	3.3305
C5···H8	3.2208	H10···H25^i^	3.2265
C6···H2	3.3074	H11···C21^i^	3.1201
C6···H4	3.2492	H11···C22^i^	2.9476
C6···H8	2.8819	H11···C23^i^	2.7948
C7···H5	2.8662	H11···C24^i^	2.8600
C7···H9	3.2514	H11···C25^i^	3.0466
C7···H11	3.2934	H11···C26^i^	3.1521
C8···H5	3.2121	H11···H22^i^	3.4705
C8···H10	3.2630	H11···H23^i^	3.2479
C9···H11	3.2784	H11···H24^i^	3.3524
C10···H8	3.2728	H11···H37C^ii^	3.0481
C11···H9	3.2680	H11···H38C^ii^	3.4828
C11···H13	2.7690	H13···C22^i^	3.1563
C12···H8	3.2843	H13···C23^i^	3.0430
C12···H10	3.2294	H13···C37^ii^	3.4537
C12···H13	2.6224	H13···C38^ii^	3.5449
C13···H2	3.0174	H13···H22^i^	3.0827
C13···H11	2.8553	H13···H23^i^	2.8696
C14···H2	2.7073	H13···H24^iii^	3.0257
C15···H19A	2.7060	H13···H37A^ii^	3.1325
C15···H19B	3.4063	H13···H37C^ii^	2.9407
C15···H19C	2.8307	H13···H38B^ii^	3.2048
C15···H20A	3.4310	H13···H38C^ii^	3.0730
C15···H20B	2.7680	H14···O3	2.6754
C15···H20C	2.8499	H14···C24^iii^	3.5794
C16···H17A	2.6979	H14···C35	3.5176
C16···H17B	3.3999	H14···C38	3.1802
C16···H17C	2.8198	H14···H24^iii^	3.0246
C16···H18A	3.4202	H14···H25^iii^	3.1952
C16···H18B	2.7461	H14···H33	3.1980
C16···H18C	2.8420	H14···H38A	2.9460
C17···H18A	2.6686	H14···H38B	2.7622
C17···H18B	3.3409	H17A···C9^x^	3.4193
C17···H18C	2.7106	H17A···C10^x^	3.1239
C17···H20B	2.5614	H17A···C11^x^	3.4394
C17···H20C	3.1232	H17A···H10^x^	3.2488
C18···H17A	3.3396	H17A···H23^i^	2.8395
C18···H17B	2.6731	H17A···H24^iii^	3.3931
C18···H17C	2.7096	H17A···H25^iii^	3.4855
C18···H19A	2.9053	H17B···C22^i^	3.3821
C18···H19C	2.7068	H17B···C23^i^	2.9814
C18···H20C	3.3551	H17B···C29^x^	3.1483
C19···H18B	2.5385	H17B···C30^x^	2.9601
C19···H18C	3.1228	H17B···H22^i^	3.1321
C19···H20A	2.6581	H17B···H23^i^	2.3248
C19···H20B	3.3434	H17B···H29^x^	2.9741
C19···H20C	2.7170	H17B···H30^x^	2.6374
C20···H17A	2.9148	H17C···C9^x^	3.4345
C20···H17C	2.7084	H17C···C29^xii^	3.0266
C20···H18C	3.3563	H17C···H9^x^	3.5047
C20···H19A	3.3428	H17C···H29^xii^	2.5097
C20···H19B	2.6698	H17C···H29^x^	3.4017
C20···H19C	2.7078	H17C···H30^x^	3.2977
C21···H23	3.2440	H18A···C22^i^	3.5314
C21···H25	3.2966	H18A···C28^x^	3.5790
C21···H33	3.3191	H18A···C29^x^	3.0783
C21···H34	2.9454	H18A···H5^xii^	3.5520
C22···H24	3.2770	H18A···H8^xii^	3.1419
C22···H34	2.6857	H18A···H22^i^	2.7350
C23···H25	3.2714	H18A···H28^x^	3.5441
C24···H22	3.2856	H18A···H29^x^	2.6193
C25···H23	3.2619	H18A···H34^i^	2.7615
C25···H28	3.2083	H18B···C3^ii^	3.4227
C26···H22	3.3037	H18B···C4^ii^	3.3475
C26···H24	3.2527	H18B···C5^xii^	3.4101
C26···H28	2.8811	H18B···H3^ii^	3.2288
C27···H25	2.8609	H18B···H4^ii^	3.1088
C27···H29	3.2550	H18B···H5^xii^	2.5229
C27···H31	3.2964	H18B···H8^xii^	3.4023
C28···H25	3.2015	H18B···H34^i^	3.5610
C28···H30	3.2594	H18C···C5^xii^	3.2483
C29···H31	3.2864	H18C···C29^xii^	3.5697
C30···H28	3.2694	H18C···C30^xii^	3.2391
C31···H29	3.2762	H18C···H5^xii^	2.6356
C31···H33	2.6998	H18C···H8^xii^	3.0879
C32···H28	3.2817	H18C···H29^xii^	3.3891
C32···H30	3.2340	H18C···H29^x^	3.0961
C32···H33	2.5860	H18C···H30^xii^	2.7643
C33···H22	3.0430	H19A···C1^ii^	3.5328
C33···H31	2.8401	H19A···C2^ii^	3.3037
C34···H22	2.7325	H19A···C3^ii^	3.0062
C35···H39A	3.3943	H19A···C4^ii^	2.9686
C35···H39B	2.7001	H19A···C5^ii^	3.2092
C35···H39C	2.8132	H19A···C6^ii^	3.4812
C35···H40A	2.7344	H19A···H3^ii^	3.3887
C35···H40B	3.4078	H19A···H4^ii^	3.3451
C35···H40C	2.8162	H19A···H38A	2.8762
C36···H37A	3.4058	H19B···C38	3.4358
C36···H37B	2.7197	H19B···C39	3.2488
C36···H37C	2.8365	H19B···C40^xiii^	3.3541
C36···H38A	2.7127	H19B···H4^xii^	3.5571
C36···H38B	3.3958	H19B···H38A	2.5937
C36···H38C	2.8100	H19B···H38C	3.4631
C37···H38A	3.3303	H19B···H39A^xiii^	3.4666
C37···H38B	2.6499	H19B···H39B	2.9822
C37···H38C	2.7018	H19B···H39C	2.6398
C37···H40A	2.5399	H19B···H40B^xiii^	2.5695
C37···H40C	3.0849	H19B···H40C^xiii^	3.2955
C38···H37A	2.6644	H19C···C4^xii^	3.1214
C38···H37B	3.3290	H19C···C4^ii^	3.4047
C38···H37C	2.6915	H19C···C5^xii^	3.1528
C38···H39B	2.8542	H19C···C5^ii^	3.4970
C38···H39C	2.6485	H19C···C40^xiii^	3.5448
C38···H40C	3.3064	H19C···H4^xii^	2.6059
C39···H38A	2.4819	H19C···H4^ii^	3.4309
C39···H38C	3.0545	H19C···H5^xii^	2.6893
C39···H40A	3.3444	H19C···H5^ii^	3.5690
C39···H40B	2.6705	H19C···H40B^xiii^	2.6852
C39···H40C	2.7152	H20A···C9^vi^	3.0849
C40···H37B	2.8996	H20A···C10^vi^	3.1275
C40···H37C	2.6953	H20A···C39	3.5219
C40···H38C	3.3073	H20A···C40^xiii^	3.5302
C40···H39A	2.6815	H20A···H9^vi^	2.8175
C40···H39B	3.3453	H20A···H10^vi^	2.8790
C40···H39C	2.7026	H20A···H39B	2.9115
B1···H13	2.5945	H20A···H39C	3.5019
B1···H17A	2.8662	H20A···H40B^xiii^	3.3125
B1···H17B	3.5240	H20A···H40C^xiii^	2.9080
B1···H18B	3.5789	H20B···H9^vi^	3.1279
B1···H19A	2.8185	H20B···H9^x^	3.4972
B1···H19B	3.4857	H20B···H10^vi^	3.1254
B2···H33	2.5578	H20C···C28^xii^	3.3390
B2···H37A	3.5330	H20C···C29^xii^	3.1325
B2···H37B	2.8836	H20C···C30^xii^	3.2835
B2···H38A	3.5819	H20C···H10^vi^	3.3305
B2···H39A	3.5239	H20C···H29^xii^	3.4322
B2···H39B	2.8724	H20C···H40B^xiii^	3.4672
B2···H40A	3.5954	H20C···H40C^xiii^	3.4191
H2···H3	2.3452	H22···O1^iv^	2.7802
H2···H14	2.0058	H22···C15^iv^	3.5198
H3···H4	2.3245	H22···C18^iv^	3.4450
H4···H5	2.3305	H22···H11^iv^	3.4705
H5···H8	2.8167	H22···H13^iv^	3.0827
H8···H9	2.3326	H22···H17B^iv^	3.1321
H9···H10	2.3327	H22···H18A^iv^	2.7350
H10···H11	2.3325	H22···H37A^v^	3.3954
H11···H13	2.3058	H22···H37C^v^	3.1823
H13···H14	2.7814	H23···O1^iv^	2.7517
H17A···H18A	3.5549	H23···C13^iv^	3.4922
H17A···H20B	2.3387	H23···C15^iv^	3.4131
H17A···H20C	3.3752	H23···C17^iv^	2.9194
H17B···H18A	2.4713	H23···C24^xiv^	3.5932
H17B···H18B	3.5581	H23···B1^iv^	3.4196
H17B···H18C	2.9835	H23···H11^iv^	3.2479
H17B···H20B	3.5293	H23···H13^iv^	2.8696
H17C···H18A	2.9753	H23···H17A^iv^	2.8395
H17C···H18C	2.5558	H23···H17B^iv^	2.3248
H17C···H20B	2.3273	H23···H24^xiv^	2.8649
H17C···H20C	2.6650	H24···N1^iii^	3.3862
H18B···H19A	2.3166	H24···C13^iii^	2.7911
H18B···H19B	3.5070	H24···C14^iii^	2.7126
H18B···H19C	2.3103	H24···C23^xiv^	3.5183
H18C···H19A	3.3732	H24···B1^iii^	3.1429
H18C···H19C	2.6708	H24···H11^iv^	3.3524
H18C···H20C	2.9358	H24···H13^iii^	3.0257
H19A···H20A	3.5480	H24···H14^iii^	3.0246
H19B···H20A	2.4559	H24···H17A^iii^	3.3931
H19B···H20B	3.5520	H24···H23^xiv^	2.8649
H19B···H20C	2.9901	H24···H24^xiv^	3.4897
H19C···H20A	2.9590	H25···O2^iii^	3.2299
H19C···H20C	2.5602	H25···C14^iii^	3.3856
H22···H23	2.3440	H25···C33^iii^	3.5184
H22···H34	2.0045	H25···B1^iii^	3.4434
H23···H24	2.3228	H25···H10^iv^	3.2265
H24···H25	2.3380	H25···H14^iii^	3.1952
H25···H28	2.8023	H25···H17A^iii^	3.4855
H28···H29	2.3329	H25···H33^iii^	3.4850
H29···H30	2.3303	H28···C9^xi^	3.3784
H30···H31	2.3407	H28···C34^iii^	3.3559
H31···H33	2.2260	H28···H8^xi^	3.2629
H33···H34	2.7762	H28···H9^xi^	2.6427
H37A···H38A	3.5468	H28···H18A^x^	3.5441
H37A···H38B	2.4544	H28···H34^iii^	3.0271
H37A···H38C	2.9837	H29···C17^viii^	3.4562
H37A···H40A	3.5092	H29···C17^x^	3.5597
H37B···H38B	3.5410	H29···C18^x^	3.2710
H37B···H38C	3.5927	H29···H8^xi^	3.3817
H37B···H40A	2.3124	H29···H17B^x^	2.9741
H37B···H40C	3.3416	H29···H17C^viii^	2.5097
H37C···H38A	3.5922	H29···H17C^x^	3.4017
H37C···H38B	2.9467	H29···H18A^x^	2.6193
H37C···H38C	2.5394	H29···H18C^viii^	3.3891
H37C···H40A	2.3229	H29···H18C^x^	3.0961
H37C···H40C	2.6265	H29···H20C^viii^	3.4322
H38A···H39A	3.4490	H29···H29^xi^	3.5140
H38A···H39B	2.2510	H30···C5	3.4040
H38A···H39C	2.2617	H30···C6	3.0763
H38C···H39B	3.3096	H30···C7	3.1137
H38C···H39C	2.5874	H30···C8	3.4874
H38C···H40C	2.8757	H30···C17^x^	3.3842
H39A···H40A	3.5637	H30···H5	3.5397
H39A···H40B	2.4788	H30···H8	3.5972
H39A···H40C	2.9976	H30···H17B^x^	2.6374
H39B···H40B	3.5624	H30···H17C^x^	3.2977
H39C···H40B	2.9623	H30···H18C^viii^	2.7643
H39C···H40C	2.5497	H31···N1	3.3851
O1···H22^i^	2.7802	H31···C1	2.7556
O1···H23^i^	2.7517	H31···C2	2.8692
O2···H25^iii^	3.2299	H31···C3	3.1833
O2···H38A	2.6201	H31···C4	3.4162
O2···H39B	2.8790	H31···C5	3.3778
O3···H2	2.5567	H31···C6	3.0548
O3···H3	3.5567	H31···H2	3.2950
O3···H14	2.6754	H33···C2	3.1050
O4···H3^v^	2.9576	H33···C3	3.3666
O4···H4^v^	3.4571	H33···H2	2.9556
O4···H8^vi^	3.4179	H33···H3	3.3831
N1···H24^iii^	3.3862	H33···H14	3.1980
N1···H31	3.3851	H33···H25^iii^	3.4850
N1···H38B^ii^	3.1651	H33···H37B^v^	3.2274
N1···H38C^ii^	2.6113	H33···H40A^v^	3.4549
N2···H37C^v^	3.3943	H34···C18^iv^	3.5729
C1···H19A^ii^	3.5328	H34···C28^iii^	3.2475
C1···H31	2.7556	H34···H18A^iv^	2.7615
C1···H38B^ii^	3.4777	H34···H18B^iv^	3.5610
C1···H38C^ii^	3.0690	H34···H28^iii^	3.0271
C2···H19A^ii^	3.3037	H34···H37A^v^	3.4395
C2···H31	2.8692	H34···H37B^v^	3.3294
C2···H33	3.1050	H34···H37C^v^	3.5750
C2···H37A	3.2809	H37A···C2	3.2809
C2···H38B	3.4697	H37A···C3	3.2319
C3···H18B^ii^	3.4227	H37A···C34^v^	3.5331
C3···H19A^ii^	3.0062	H37A···H2	2.7348
C3···H31	3.1833	H37A···H3	2.6147
C3···H33	3.3666	H37A···H13^ii^	3.1325
C3···H37A	3.2319	H37A···H22^v^	3.3954
C3···H40A^v^	3.3213	H37A···H34^v^	3.4395
C4···H4^vii^	3.5083	H37B···C33^v^	3.0667
C4···H5^vii^	3.2378	H37B···C34^v^	2.9875
C4···H18B^ii^	3.3475	H37B···B2^v^	3.2813
C4···H19A^ii^	2.9686	H37B···H3	3.3418
C4···H19C^viii^	3.1214	H37B···H3^v^	3.4791
C4···H19C^ii^	3.4047	H37B···H33^v^	3.2274
C4···H31	3.4162	H37B···H34^v^	3.3294
C4···H40A^v^	3.3780	H37B···H37B^v^	3.2079
C4···H40B^v^	3.1340	H37C···N2^v^	3.3943
C5···H4^vii^	3.1527	H37C···C11^ii^	3.4647
C5···H18B^viii^	3.4101	H37C···C21^v^	3.4444
C5···H18C^viii^	3.2483	H37C···C22^v^	3.3871
C5···H19A^ii^	3.2092	H37C···C33^v^	3.3930
C5···H19C^viii^	3.1528	H37C···C34^v^	3.5375
C5···H19C^ii^	3.4970	H37C···H11^ii^	3.0481
C5···H30	3.4040	H37C···H13^ii^	2.9407
C5···H31	3.3778	H37C···H22^v^	3.1823
C6···H19A^ii^	3.4812	H37C···H34^v^	3.5750
C6···H30	3.0763	H38A···O2	2.6201
C6···H31	3.0548	H38A···C14	3.2875
C6···H38C^ii^	3.4422	H38A···C16	3.4510
C7···H30	3.1137	H38A···C19	3.0622
C7···H38C^ii^	3.2615	H38A···B1	3.1009
C7···H39A^ix^	3.4810	H38A···H14	2.9460
C8···H30	3.4874	H38A···H19A	2.8762
C8···H39A^ix^	2.8891	H38A···H19B	2.5937
C9···H17A^x^	3.4193	H38B···N1^ii^	3.1651
C9···H17C^x^	3.4345	H38B···C1^ii^	3.4777
C9···H20A^ix^	3.0849	H38B···C2	3.4697
C9···H28^xi^	3.3784	H38B···C13^ii^	3.0944
C9···H39A^ix^	3.3302	H38B···C14	3.1916
C9···H39B^ix^	3.5354	H38B···H2	2.5925
C10···H17A^x^	3.1239	H38B···H13^ii^	3.2048
C10···H20A^ix^	3.1275	H38B···H14	2.7622
C10···H40C^ii^	3.5263	H38C···N1^ii^	2.6113
C11···H17A^x^	3.4394	H38C···C1^ii^	3.0690
C11···H37C^ii^	3.4647	H38C···C6^ii^	3.4422
C11···H38C^ii^	3.2691	H38C···C7^ii^	3.2615
C11···H40C^ii^	3.5352	H38C···C11^ii^	3.2691
C12···H38C^ii^	2.7261	H38C···C12^ii^	2.7261
C13···H23^i^	3.4922	H38C···C13^ii^	3.0653
C13···H24^iii^	2.7911	H38C···H11^ii^	3.4828
C13···H38B^ii^	3.0944	H38C···H13^ii^	3.0730
C13···H38C^ii^	3.0653	H38C···H19B	3.4631
C14···H24^iii^	2.7126	H39A···C7^vi^	3.4810
C14···H25^iii^	3.3856	H39A···C8^vi^	2.8891
C14···H38A	3.2875	H39A···C9^vi^	3.3302
C14···H38B	3.1916	H39A···H8^vi^	2.7319
C15···H22^i^	3.5198	H39A···H9^vi^	3.4756
C15···H23^i^	3.4131	H39A···H19B^xiii^	3.4666
C16···H38A	3.4510	H39A···H39C^xiii^	2.8638
C17···H23^i^	2.9194	H39B···O2	2.8790
C17···H29^xii^	3.4562	H39B···C9^vi^	3.5354
C17···H29^x^	3.5597	H39B···H8^vi^	3.3861
C17···H30^x^	3.3842	H39B···H9^vi^	3.2565
C18···H5^xii^	3.0086	H39B···H19B	2.9822
C18···H8^xii^	3.3972	H39B···H20A	2.9115
C18···H22^i^	3.4450	H39C···C19	3.5707
C18···H29^x^	3.2710	H39C···C39^xiii^	3.2368
C18···H34^i^	3.5729	H39C···H19B	2.6398
C19···H4^xii^	3.4437	H39C···H20A	3.5019
C19···H5^xii^	3.5579	H39C···H39A^xiii^	2.8638
C19···H38A	3.0622	H39C···H39C^xiii^	2.7173
C19···H39C	3.5707	H40A···C3^v^	3.3213
C19···H40B^xiii^	3.0629	H40A···C4^v^	3.3780
C20···H9^vi^	3.4233	H40A···C33^v^	3.5877
C20···H10^vi^	3.2907	H40A···H3^v^	2.9959
C20···H40C^xiii^	3.5942	H40A···H4^v^	3.0916
C21···H11^iv^	3.1201	H40A···H33^v^	3.4549
C21···H37C^v^	3.4444	H40B···C4^v^	3.1340
C22···H11^iv^	2.9476	H40B···C19^xiii^	3.0629
C22···H13^iv^	3.1563	H40B···H3^v^	3.4447
C22···H17B^iv^	3.3821	H40B···H4^v^	2.4566
C22···H18A^iv^	3.5314	H40B···H19B^xiii^	2.5695
C22···H37C^v^	3.3871	H40B···H19C^xiii^	2.6852
C23···H11^iv^	2.7948	H40B···H20A^xiii^	3.3125
C23···H13^iv^	3.0430	H40B···H20C^xiii^	3.4672
C23···H17B^iv^	2.9814	H40C···C10^ii^	3.5263
C23···H24^xiv^	3.5183	H40C···C11^ii^	3.5352
C24···H11^iv^	2.8600	H40C···C20^xiii^	3.5942
C24···H14^iii^	3.5794	H40C···H19B^xiii^	3.2955
C24···H23^xiv^	3.5932	H40C···H20A^xiii^	2.9080
C25···H10^iv^	3.2043	H40C···H20C^xiii^	3.4191
C25···H11^iv^	3.0466		
			
C15—O1—B1	107.1 (2)	C2—C3—H3	119.303
C16—O2—B1	106.48 (19)	C4—C3—H3	119.295
C35—O3—B2	107.2 (2)	C3—C4—H4	119.591
C36—O4—B2	107.2 (3)	C5—C4—H4	119.600
C1—N1—C12	108.38 (17)	C4—C5—H5	120.374
C1—N1—C13	128.6 (3)	C6—C5—H5	120.367
C12—N1—C13	122.97 (19)	C7—C8—H8	120.545
C21—N2—C32	107.63 (17)	C9—C8—H8	120.546
C21—N2—C33	129.5 (3)	C8—C9—H9	119.646
C32—N2—C33	122.8 (2)	C10—C9—H9	119.648
N1—C1—C2	130.62 (19)	C9—C10—H10	119.380
N1—C1—C6	108.2 (3)	C11—C10—H10	119.379
C2—C1—C6	121.11 (19)	C10—C11—H11	120.911
C1—C2—C3	117.8 (2)	C12—C11—H11	120.902
C2—C3—C4	121.4 (3)	N1—C13—H13	115.810
C3—C4—C5	120.8 (2)	C14—C13—H13	115.792
C4—C5—C6	119.3 (3)	C13—C14—H14	119.238
C1—C6—C5	119.6 (3)	B1—C14—H14	119.229
C1—C6—C7	107.47 (18)	C15—C17—H17A	109.471
C5—C6—C7	133.0 (3)	C15—C17—H17B	109.472
C6—C7—C8	133.36 (19)	C15—C17—H17C	109.469
C6—C7—C12	107.1 (2)	H17A—C17—H17B	109.473
C8—C7—C12	119.5 (3)	H17A—C17—H17C	109.467
C7—C8—C9	118.9 (2)	H17B—C17—H17C	109.476
C8—C9—C10	120.7 (3)	C15—C18—H18A	109.475
C9—C10—C11	121.2 (3)	C15—C18—H18B	109.472
C10—C11—C12	118.19 (19)	C15—C18—H18C	109.471
N1—C12—C7	108.7 (2)	H18A—C18—H18B	109.464
N1—C12—C11	129.82 (18)	H18A—C18—H18C	109.477
C7—C12—C11	121.4 (3)	H18B—C18—H18C	109.469
N1—C13—C14	128.4 (3)	C16—C19—H19A	109.472
C13—C14—B1	121.5 (3)	C16—C19—H19B	109.469
O1—C15—C16	101.87 (17)	C16—C19—H19C	109.475
O1—C15—C17	107.33 (17)	H19A—C19—H19B	109.473
O1—C15—C18	107.9 (2)	H19A—C19—H19C	109.468
C16—C15—C17	112.8 (3)	H19B—C19—H19C	109.472
C16—C15—C18	115.13 (19)	C16—C20—H20A	109.472
C17—C15—C18	111.0 (2)	C16—C20—H20B	109.472
O2—C16—C15	102.30 (17)	C16—C20—H20C	109.473
O2—C16—C19	106.33 (16)	H20A—C20—H20B	109.472
O2—C16—C20	108.41 (19)	H20A—C20—H20C	109.471
C15—C16—C19	113.0 (2)	H20B—C20—H20C	109.468
C15—C16—C20	115.62 (19)	C21—C22—H22	121.026
C19—C16—C20	110.3 (2)	C23—C22—H22	121.021
N2—C21—C22	130.71 (19)	C22—C23—H23	119.208
N2—C21—C26	108.4 (3)	C24—C23—H23	119.202
C22—C21—C26	120.8 (2)	C23—C24—H24	119.621
C21—C22—C23	118.0 (2)	C25—C24—H24	119.608
C22—C23—C24	121.6 (3)	C24—C25—H25	120.600
C23—C24—C25	120.8 (3)	C26—C25—H25	120.573
C24—C25—C26	118.8 (3)	C27—C28—H28	120.473
C21—C26—C25	120.0 (3)	C29—C28—H28	120.476
C21—C26—C27	107.57 (19)	C28—C29—H29	119.737
C25—C26—C27	132.3 (2)	C30—C29—H29	119.731
C26—C27—C28	133.4 (2)	C29—C30—H30	119.240
C26—C27—C32	107.0 (2)	C31—C30—H30	119.235
C28—C27—C32	119.5 (3)	C30—C31—H31	121.147
C27—C28—C29	119.1 (2)	C32—C31—H31	121.165
C28—C29—C30	120.5 (3)	N2—C33—H33	114.977
C29—C30—C31	121.5 (3)	C34—C33—H33	114.962
C30—C31—C32	117.7 (2)	C33—C34—H34	119.747
N2—C32—C27	109.3 (3)	B2—C34—H34	119.740
N2—C32—C31	129.05 (19)	C35—C37—H37A	109.473
C27—C32—C31	121.7 (3)	C35—C37—H37B	109.468
N2—C33—C34	130.1 (3)	C35—C37—H37C	109.469
C33—C34—B2	120.5 (3)	H37A—C37—H37B	109.473
O3—C35—C36	101.98 (18)	H37A—C37—H37C	109.473
O3—C35—C37	106.51 (16)	H37B—C37—H37C	109.472
O3—C35—C38	108.9 (2)	C35—C38—H38A	109.469
C36—C35—C37	114.3 (3)	C35—C38—H38B	109.474
C36—C35—C38	113.89 (18)	C35—C38—H38C	109.467
C37—C35—C38	110.5 (2)	H38A—C38—H38B	109.471
O4—C36—C35	102.74 (19)	H38A—C38—H38C	109.475
O4—C36—C39	106.7 (2)	H38B—C38—H38C	109.471
O4—C36—C40	109.1 (3)	C36—C39—H39A	109.468
C35—C36—C39	113.0 (3)	C36—C39—H39B	109.474
C35—C36—C40	114.0 (3)	C36—C39—H39C	109.473
C39—C36—C40	110.7 (3)	H39A—C39—H39B	109.465
O1—B1—O2	113.12 (17)	H39A—C39—H39C	109.464
O1—B1—C14	124.6 (3)	H39B—C39—H39C	109.483
O2—B1—C14	122.3 (3)	C36—C40—H40A	109.473
O3—B2—O4	112.9 (2)	C36—C40—H40B	109.468
O3—B2—C34	123.1 (3)	C36—C40—H40C	109.475
O4—B2—C34	124.0 (3)	H40A—C40—H40B	109.469
C1—C2—H2	121.107	H40A—C40—H40C	109.476
C3—C2—H2	121.095	H40B—C40—H40C	109.466
			
C15—O1—B1—O2	−8.9 (3)	C8—C7—C12—N1	179.3 (2)
C15—O1—B1—C14	169.59 (18)	C8—C7—C12—C11	−0.5 (4)
B1—O1—C15—C16	23.79 (19)	C12—C7—C8—C9	0.7 (4)
B1—O1—C15—C17	−94.9 (2)	C7—C8—C9—C10	0.1 (4)
B1—O1—C15—C18	145.40 (17)	C8—C9—C10—C11	−1.1 (4)
C16—O2—B1—O1	−11.6 (3)	C9—C10—C11—C12	1.3 (4)
C16—O2—B1—C14	169.92 (17)	C10—C11—C12—N1	179.8 (2)
B1—O2—C16—C15	25.37 (19)	C10—C11—C12—C7	−0.5 (4)
B1—O2—C16—C19	−93.4 (2)	N1—C13—C14—B1	−177.40 (17)
B1—O2—C16—C20	147.99 (17)	C13—C14—B1—O1	−5.3 (4)
C35—O3—B2—O4	10.1 (2)	C13—C14—B1—O2	173.09 (19)
C35—O3—B2—C34	−169.04 (17)	O1—C15—C16—O2	−29.61 (19)
B2—O3—C35—C36	−23.38 (17)	O1—C15—C16—C19	84.30 (16)
B2—O3—C35—C37	96.74 (19)	O1—C15—C16—C20	−147.18 (14)
B2—O3—C35—C38	−144.05 (16)	C17—C15—C16—O2	85.14 (18)
C36—O4—B2—O3	9.1 (3)	C17—C15—C16—C19	−160.95 (14)
C36—O4—B2—C34	−171.82 (18)	C17—C15—C16—C20	−32.4 (2)
B2—O4—C36—C35	−23.0 (2)	C18—C15—C16—O2	−146.09 (17)
B2—O4—C36—C39	96.1 (3)	C18—C15—C16—C19	−32.2 (3)
B2—O4—C36—C40	−144.28 (18)	C18—C15—C16—C20	96.3 (2)
C1—N1—C12—C7	1.7 (3)	N2—C21—C22—C23	−178.07 (16)
C1—N1—C12—C11	−178.5 (2)	N2—C21—C26—C25	179.51 (14)
C12—N1—C1—C2	176.8 (2)	N2—C21—C26—C27	1.52 (19)
C12—N1—C1—C6	−1.4 (3)	C22—C21—C26—C25	2.2 (3)
C1—N1—C13—C14	−19.3 (4)	C22—C21—C26—C27	−175.78 (15)
C13—N1—C1—C2	−3.7 (4)	C26—C21—C22—C23	−1.4 (3)
C13—N1—C1—C6	178.07 (18)	C21—C22—C23—C24	−0.1 (3)
C12—N1—C13—C14	160.09 (19)	C22—C23—C24—C25	1.0 (3)
C13—N1—C12—C7	−177.80 (17)	C23—C24—C25—C26	−0.2 (3)
C13—N1—C12—C11	2.0 (4)	C24—C25—C26—C21	−1.3 (3)
C21—N2—C32—C27	2.11 (19)	C24—C25—C26—C27	176.06 (18)
C21—N2—C32—C31	−177.67 (16)	C21—C26—C27—C28	178.48 (17)
C32—N2—C21—C22	174.71 (16)	C21—C26—C27—C32	−0.22 (19)
C32—N2—C21—C26	−2.23 (18)	C25—C26—C27—C28	0.8 (4)
C21—N2—C33—C34	−6.8 (4)	C25—C26—C27—C32	−177.86 (19)
C33—N2—C21—C22	−8.4 (3)	C26—C27—C28—C29	−178.42 (18)
C33—N2—C21—C26	174.70 (16)	C26—C27—C32—N2	−1.2 (2)
C32—N2—C33—C34	169.73 (17)	C26—C27—C32—C31	178.64 (15)
C33—N2—C32—C27	−175.07 (14)	C28—C27—C32—N2	179.92 (16)
C33—N2—C32—C31	5.1 (3)	C28—C27—C32—C31	−0.3 (3)
N1—C1—C2—C3	179.6 (2)	C32—C27—C28—C29	0.1 (3)
N1—C1—C6—C5	−179.67 (17)	C27—C28—C29—C30	0.4 (3)
N1—C1—C6—C7	0.6 (3)	C28—C29—C30—C31	−0.8 (3)
C2—C1—C6—C5	1.9 (4)	C29—C30—C31—C32	0.7 (3)
C2—C1—C6—C7	−177.86 (19)	C30—C31—C32—N2	179.62 (17)
C6—C1—C2—C3	−2.3 (4)	C30—C31—C32—C27	−0.1 (3)
C1—C2—C3—C4	0.9 (4)	N2—C33—C34—B2	176.99 (17)
C2—C3—C4—C5	0.9 (4)	C33—C34—B2—O3	9.9 (3)
C3—C4—C5—C6	−1.4 (4)	C33—C34—B2—O4	−169.14 (19)
C4—C5—C6—C1	0.0 (4)	O3—C35—C36—O4	27.75 (19)
C4—C5—C6—C7	179.7 (2)	O3—C35—C36—C39	−86.81 (19)
C1—C6—C7—C8	179.7 (3)	O3—C35—C36—C40	145.62 (15)
C1—C6—C7—C12	0.5 (3)	C37—C35—C36—O4	−86.7 (2)
C5—C6—C7—C8	−0.0 (5)	C37—C35—C36—C39	158.70 (17)
C5—C6—C7—C12	−179.2 (3)	C37—C35—C36—C40	31.1 (3)
C6—C7—C8—C9	−178.5 (3)	C38—C35—C36—O4	144.85 (17)
C6—C7—C12—N1	−1.4 (3)	C38—C35—C36—C39	30.3 (3)
C6—C7—C12—C11	178.84 (18)	C38—C35—C36—C40	−97.3 (2)

Symmetry codes: (i) *x*+1, *y*, *z*; (ii) −*x*+2, −*y*+2, −*z*; (iii) −*x*+1, −*y*+2, −*z*+1; (iv) *x*−1, *y*, *z*; (v) −*x*+1, −*y*+2, −*z*; (vi) *x*, *y*−1, *z*; (vii) −*x*+1, −*y*+3, −*z*; (viii) *x*−1, *y*+1, *z*; (ix) *x*, *y*+1, *z*; (x) −*x*+2, −*y*+2, −*z*+1; (xi) −*x*+1, −*y*+3, −*z*+1; (xii) *x*+1, *y*−1, *z*; (xiii) −*x*+2, −*y*+1, −*z*; (xiv) −*x*, −*y*+2, −*z*+1.

## References

[bb1] Altomare, A., Cascarano, G., Giacovazzo, C., Guagliardi, A., Burla, M. C., Polidori, G. & Camalli, M. (1994). *J. Appl. Cryst.* **27**, 435.

[bb2] Clark, J. S., Freeman, R. P., Cacho, M., Thomas, A. W., Swallow, S. & Wilson, C. (2004). *Tetrahedron Lett.* **45**, 8639–8642.

[bb3] Farrugia, L. J. (1997). *J. Appl. Cryst.* **30**, 565.

[bb4] Geier, M. J., Vogels, C. M., Decken, A. & Westcott, S. A. (2009). *J. Organomet. Chem.* **694**, 3154–3159.

[bb5] Rigaku (1999). *NUMABS* Rigaku Corporation, Tokyo, Japan.

[bb6] Rigaku (2008). *CrystalClear* Rigaku Corporation, Tokyo, Japan.

[bb7] Rigaku (2010). *CrystalStructure* Rigaku Corporation, Tokyo, Japan.

[bb8] Sheldrick, G. M. (2008). *Acta Cryst.* A**64**, 112–122. 10.1107/S0108767307043930 18156677

[bb9] Tian, X., Shi, W., Shen, K., Li, C., Lin, J., Che, Y. & Zhang, P. (2006). *J. Organomet. Chem.* **691**, 994–1006.

[bb10] Tsutsui, K., Hirotsu, K., Umesaki, M., Kurahashi, M., Shimada, A. & Higuchi, T. (1976). *Acta Cryst.* B**32**, 3049–3053.

